# The prediction for development of COVID-19 in global major epidemic areas through empirical trends in China by utilizing state transition matrix model

**DOI:** 10.1186/s12879-020-05417-5

**Published:** 2020-09-29

**Authors:** Zhong Zheng, Ke Wu, Zhixian Yao, Xinyi Zheng, Junhua Zheng, Jian Chen

**Affiliations:** 1 Department of Urology, Shanghai General Hospital, Shanghai Jiao Tong University, School of Medicine, Shanghai, China; 2 Department of Pharmacy, Huashan Hospital, Fudan University, Shanghai, China; 3 School of Pharmacy, Fudan University, Shanghai, China; 4 CreditWise Technology Company Limited, Floor 4-5, Section B, Building 1 Tianfu 5th Ave., Chengdu Hi-tech Zone, Chengdu, 610041 China; 5 Caixin Insight Group, Beijing, China; 6 MSCI, Shanghai, China

**Keywords:** Coronavirus disease 2019, Novel coronavirus pneumonia, Prediction, The state transition matrix model, Inflection point

## Abstract

**Background:**

Since pneumonia caused by coronavirus disease 2019 (COVID-19) broke out in Wuhan, Hubei province, China, tremendous infected cases has risen all over the world attributed to its high transmissibility. We aimed to mathematically forecast the inflection point (IFP) of new cases in South Korea, Italy, and Iran, utilizing the transcendental model from China.

**Methods:**

Data from reports released by the National Health Commission of the People’s Republic of China (Dec 31, 2019 to Mar 5, 2020) and the World Health Organization (Jan 20, 2020 to Mar 5, 2020) were extracted as the training set and the data from Mar 6 to 9 as the validation set. New close contacts, newly confirmed cases, cumulative confirmed cases, non-severe cases, severe cases, critical cases, cured cases, and death were collected and analyzed. We analyzed the data above through the State Transition Matrix model.

**Results:**

The optimistic scenario (non-Hubei model, daily increment rate of − 3.87%), the cautiously optimistic scenario (Hubei model, daily increment rate of − 2.20%), and the relatively pessimistic scenario (adjustment, daily increment rate of − 1.50%) were inferred and modeling from data in China. The IFP of time in South Korea would be Mar 6 to 12, Italy Mar 10 to 24, and Iran Mar 10 to 24. The numbers of cumulative confirmed patients will reach approximately 20 k in South Korea, 209 k in Italy, and 226 k in Iran under fitting scenarios, respectively. However, with the adoption of different diagnosis criteria, the variation of new cases could impose various influences in the predictive model. If that happens, the IFP of increment will be earlier than predicted above.

**Conclusion:**

The end of the pandemic is still inapproachable, and the number of confirmed cases is still escalating. With the augment of data, the world epidemic trend could be further predicted, and it is imperative to consummate the assignment of global medical resources to curb the development of COVID-19.

## Background

Since the first case of novel coronavirus pneumonia (NCP), caused by coronavirus disease 2019 (COVID-19), occurred in Wuhan, Hubei Province, China, the dreadful epidemic broke out during Dec 2019 to Mar 2020 under the pace of Chinese Spring Festival [1]. With the untiring efforts of the people and the selfless dedication of medical staff, a total of 59,897 cured patients were discharged [2]. By 24:00 on Mar 9, China has accumulated a total of 80,754 confirmed cases (including 4794 severe cases) and 3136 dead cases [3]. However, in January, when the large-scale outbreak in China began, the disease initiated to spread to other parts of the world [4, 5]. Up to Mar 9, a total of 7382 cases were confirmed in South Korea, 7375 cases in Italy, and 6566 cases in Iran [6].

Similar to another coronavirus (CoV) —SARS-CoV— COVID-19 is an RNA virus that contains particular spike proteins conjugating with angiotensin-converting enzyme 2 (ACE2) that widely expressed in different human tissues [7]. However, its doughty transmissibility in the community has a strong correlation to reasons such as long incubation period, mild early symptoms, and the like [8, 9]. Even though some studies have proved that Remdesivir designed for the Ebola virus may have a promising effect on COVID-19 [10], the kernel strategies for the prevention and treatment of NCP are still effective quarantine as in the case of SARS [11]. After the implementation of strict isolation methods, the most significant goal is to predict the arrival of the peak and inflection point (IFP) of new cases of NCP so that administrative departments can modify current strategies.

Based on the previous data, we analyzed the epidemic situation in Hubei Province [12]. After the validation of the model in different datasets, we were able to analyze the world epidemic trends, and predict the arrival of peaks and IFPs of newly confirmed cases and provide references for NCP prevention and control strategies in various countries.

## Method

### Study population, data collection, and analysis

Data from reports, including medical observation, close contacts, confirmed cases, severe cases, critical cases, cured cases and death data and corresponding information, released by the Health Commission of Hubei Province (HCHP) (Dec 31, 2019 to Feb 8, 2020) were extracted as the training set. Primarily, the arrival of the IFP of new cases and epidemic trends in Hubei were deduced and testified in the validation set, whose data were extracted from HCHP (Feb 9, 2020 to Mar 5, 2020). Subsequently, another training set consisting of the data from the National Health Commission of the People’s Republic of China (NHC) and the World Health Organization (WHO) (Jan 20, 2020 to Mar 5, 2020) were established. Eventually, the data, including cumulative confirmed cases, cumulative cured cases, death data and corresponding information, from NHC and WHO (Mar 6 to 9, 2020) were collected and constructed the validation set. The data period starts from Dec 31, 2019 to Mar 9, 2020. Data is updated on the daily basis. All data were analyzed using Microsoft Excel (Microsoft Office 2016) and R studio (R Foundation for Statistical Computing, Vienna, Austria). The world epidemic situation was performed using the *nCov2019* package of R [13]. Histogram was obtained using the *ggplot2* packages of R.

### State transition matrix model

State transition matrix (STM) modeling is a well-regarded approach widely applied in clinical decision analysis based on computer simulation. For estimating the IFP of newly confirmed cases and the scale of cumulative cases in the globe in subsequent days, we chose the Markov model cohort simulation.

### Parameter selection and estimate

**Fig. 1:**
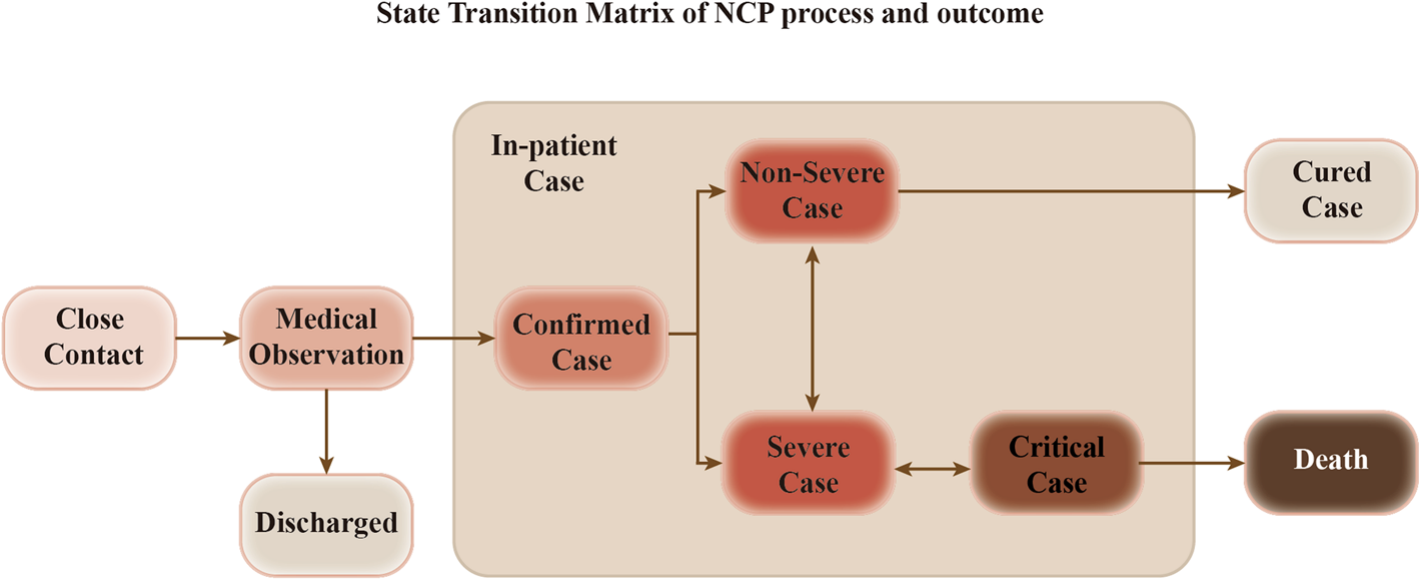
Process and outcome of the State Transition Matrix establishment when a Close Contact develops into the state of Medical Observation

In order to estimate the risk metrics (infectivity, severity, lethality) of the NCP, we build a STM model as showing in the figure (Fig. 1).

We define the states in this model. Medical Observation (MO) is the close contact of confirmed cases and put into medical observation. In the subsequent days, outcome could be any of the three: confirmed cases, discharged without COVID-19 infection, or stay in MO. Discharge (Disc) is a terminal state for a close contact, until he or she becomes another incident of close contact again. Infected is an intermediate state, where the patient becomes a confirmed infected case. The outcome is binary: severe, or non-severe. And the outcome is revealed immediately. Non-Severe Case (NS) is the patient also has three possible outcomes in the next day: cure, severe case, or stay in non-severe case. Severe Case (S), the patient has three possible outcomes in the next day: critical case, non-severe case, or stay in severe case. Critical Case (Cr), the patient has three possible outcomes in the next day: cured case, severe case, or stay in critical case. Cured Case (Cu) and Death (D) are also the terminal states for the patient. So, at any moment, we can identify the close contact or patient’s state by utilizing a state vector, defined as the following:

$$ \mathrm{V}=\left[ MO\kern1em Disc\kern1em NS\kern1em S\kern1em Cr\kern1em Cu\kern1em D\right], $$

Where each element of the vector (V) stands for one state in the same sequentially arranged order as mentioned above. Please note that the comfirmed itself is not an independent state, since the outcome is revealed instantaneously, so we combine confirmed case with Non-Severe, Severe, and Critical cases.

For each person, the state vector can only have one element with value of 1, and the other elements all have value of zero. For example, if a patient is currently in state “Severe Case”, the state vector for him is ‘[0 0 0 1 0 0 0]’. The next day, his state vector could become either ‘[0 0 0 0 1 0 0]’ (Critical Case), ‘[0 0 1 0 0 0 0]’ (Non-Severe Case) or stay the same.

For the sample population, the state vector is defined as the count of people in each state. For example, if there are 100 patients being treated today, out of which 10 are critical, and 90 are severe. The state vector for this sample population is [0 0 0 90 10 0 0]’.

Let’s define the STM as the following:

$$ \mathrm{TransMatrix}=\left[{t}_{i,j}\right] $$

Where

$$ {t}_{i,j}= daily\ transitional\ probability\ from\ state\ i\  to\ state\ j $$

Suppose we have a state vector V(t) for a sample population at time t, how do we predict the state vector V(t + 1) in the next day?

Apply simple linear algebra, we can get the following equation:

$$ \mathrm{V}\left(\mathrm{t}+1\right)=\mathrm{TransMatrix}\ast \mathrm{V}\left(\mathrm{t}\right) $$

Since the head count of a certain state comes from itself, all other possible transitions into the state (e.g. S has three possible income states, MO, NS, and Cr), minus the outcome states (NS, and Cr).

If we want to predict for N period, the equation becomes the following:

$$ \mathrm{V}\left(\mathrm{t}+\mathrm{N}\right)={\mathrm{TransMatrix}}^N\ast \mathrm{V}\left(\mathrm{t}\right) $$

If the population is limited and the transition matrix is stationary, the above formula will be sufficient in predicting all future outcomes. In our case, the population is not fixed, so we need to introduce the additional input into the population: new close contacts (NCC).

Every day, new close contacts are added to the medical observation pool, as people already in the pool will gradually be discharged or confirmed of infection.

$$ \mathrm{MO}\left(\mathrm{t}+1\right)=\mathrm{MO}\left(\mathrm{t}\right)+\mathrm{NCC}\left(\mathrm{t}+1\right)-\mathrm{Disc}\left(\mathrm{t}+1\right)-\mathrm{Confirmed}\left(\mathrm{t}+1\right) $$

Also, we assume NCC will gradually decay as quarantine measures are put into effect.

$$ \mathrm{NCC}\left(\mathrm{t}+\mathrm{N}\right)={\mathrm{e}}^{Increment\ast N}\ast \mathrm{NCC}\left(\mathrm{t}\right) $$

Using this STM model, we will be able to predict when the inflection peak time as well as IFP of newly confirmed cases (the maximum open infection cases) in Hubei Province or non-Hubei will occur. Moreover, after verifying this matrix model in China, it could be utilized to evaluate the world epidemic development especially in the major epidemic areas.

Although there is an intermediate state during the above hospitalization: severe cases (the new standard is broken down into mild and normal), critical cases (which can also be divided into general critical and critical), due to the lack of intermediate state transfer probability, we combine the entire hospital period into a in-patient state, for the sake of keeping the model simple. This minimizes the need for only the following five parameters.

- Increment of New Close Contacts (NCC), defined as ln (NCC(t)/NCC(t-1));
- Discharge Rate from Medical Observation (MO), defined as Discharged(t)/MO(t-1)
- Transitional Probability of Medical Observation - > Confirmed cases, defined as Newly confirmed cases (t)/MO(t-1)
- Transitional Probability of Treatment - > Death, defined as New Death Incidents(t) / Treatment(t-1)
- Transitional Probability of Treatment - > Cured, defined as New Cured Incidents (t) / Treatment(t-1)

In order to estimate the count of open non-severe cases, severe cases, and critical cases, we need three more parameters:

- Ratio of Non-Severe Cases
- Ratio of Severe Cases
- Ratio of Critical Cases

### Scenario setup and prediction

After validation of the STM model in Hubei Province, we set up three different scenarios derived from China for matching and fitting the major epidemic areas comprising South Korea, Italy, and Iran, in order to control for model error, including optimistic scenario, cautiously optimistic scenario, and relatively pessimistic scenario (Table 1).

**Table 1:** Scenarios for the prediction of outside China

Scenario	S1	S2	S3
Minimum Inc	−30%	−20%	−5%
Daily Inc	−3.87%	−2.20%	−1.50%

S1: optimistic scenario; S2: cautiously optimistic scenario; S3: relatively pessimistic scenario; *Inc.* Increment

## Results

### The situation of Hubei Province, China, and the historical prediction model verification of Hubei Province in the beginning of march

**Fig. 2:**
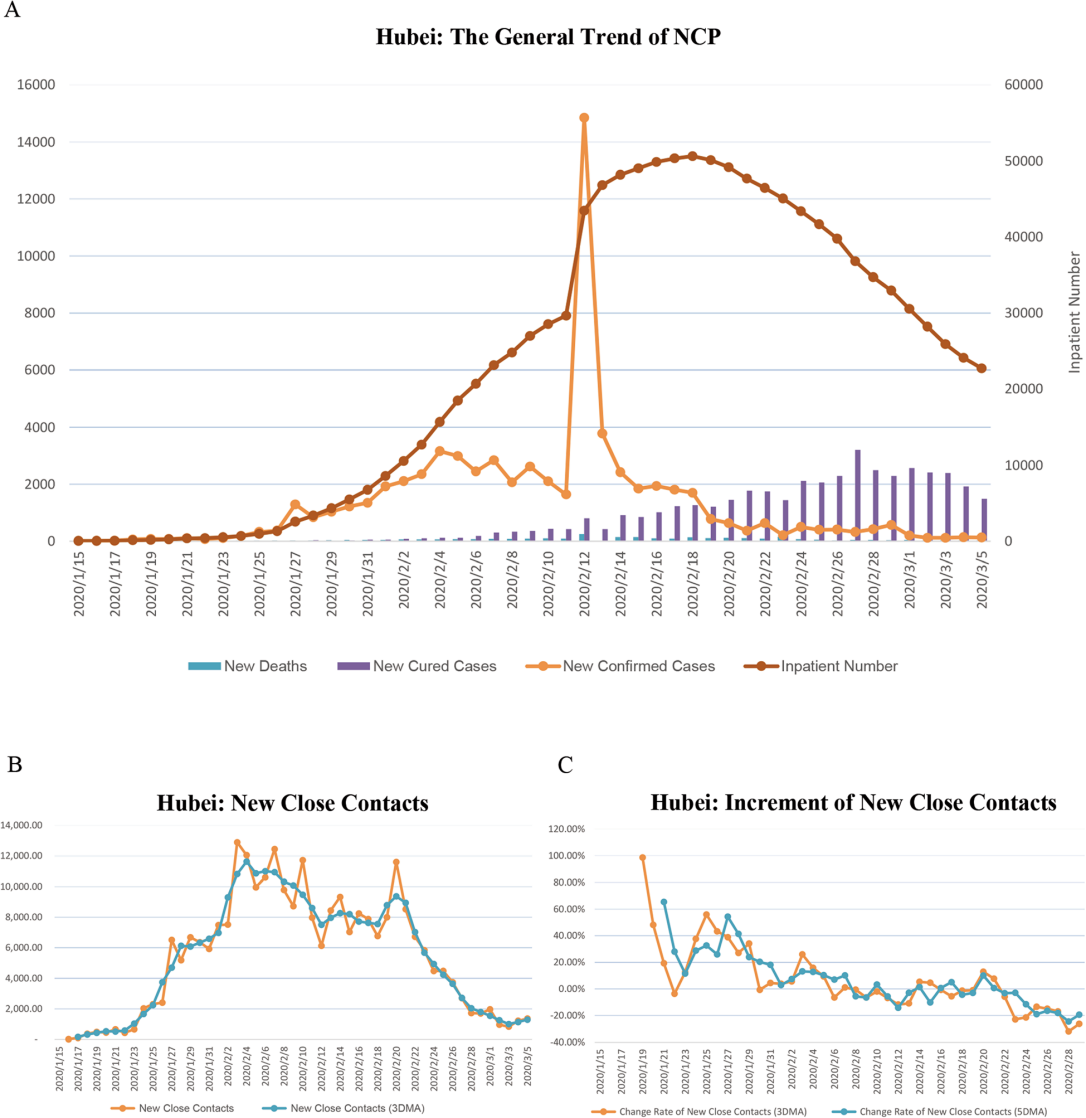
Epidemic trend in Hubei. **a** The epidemic situation and general trend in Hubei Province, including new deaths, new cured cases, newly confirmed cases, and in-patient number from Jan 15, 2020 to Mar 5, 2020. **b** The trend of new close contacts in Hubei Province from Jan 18, 2020 to Mar 5, 2020. **c** The increment of new close contacts in Hubei Province from Jan 18, 2020 to Mar 5, 2020. 3DMA: 3-day moving average; 5DMA: 5-day moving average

According to the data of NHC [14], as of Mar 5, there were 67,592 cumulative confirmed cases, 41,966 cumulative cured cases, 126 newly confirmed cases, 29 new deaths, and 1478 new cured cases, and 19,758 in-patient cases in Hubei Province (Fig. 2a). The number of new close contacts in Hubei Province has gradually decreased, and the cumulative number of close contacts is currently 271,959 (Fig. 2b). The increment of new close contacts has crossed the IFP (Fig. 2c). Based on data from Dec 31, 2019 to Feb 8, 2020 in Hubei Province, we built a prediction model through the STM model, and the cautiously optimistic scenario could consummately predict the arrival of the IFP and several peak dates in Hubei (Table 2), which undoubtedly validate the predictive efficacy of this mathematic model.

**Table 2:** Training set and Validation Set of the Epidemic Trend in Hubei Province

	Actual	Forecast					
Key Metrics		S1		S2		S3	
Inc of Confirmed Cases	−9.0%	− 10%	−10%	− 5%	−5%	−1%	− 1%
MO Release Rate	16.0%	17.0%	10.50%	17.0%	10.50%	17.0%	10.50%
Peak of Active Cases	50,633	39,612	47,148	44,082	55,150	62,041	85,502
Peak Date	2020/2/16	2020/2/23	2020/2/28	2020/3/1	2020/3/7	2020/4/6	2020/4/14
Peak of Severe Cases	9289	5753	6845	6400	8004	9000	12,402
Peak Date	2020/2/16	2020/2/23	2020/2/28	2020/3/1	2020/3/7	2020/4/6	2020/4/14
Peak of Critical Cases	2492	1786	2124	1986	2484	2793	3849
Peak Case	2020/2/21	2020/2/23	2020/2/28	2020/3/1	2020/3/7	2020/4/6	2020/4/14
Total Cases at Feb. End	66,907	54,189	64,064	60,192	71,596	68,045	81,284

The actual data were extracted from HCHP, and the forecast data in the three scenarios were deduced by the STM model based on the data before Feb 9, 2020. S1: optimistic scenario; S2: cautiously optimistic scenario; S3: relatively pessimistic scenario; *MO* Medical observation, *Inc.* Increment

### Epidemic situation in training set and the epidemic trend fitting model

**Fig. 3:**
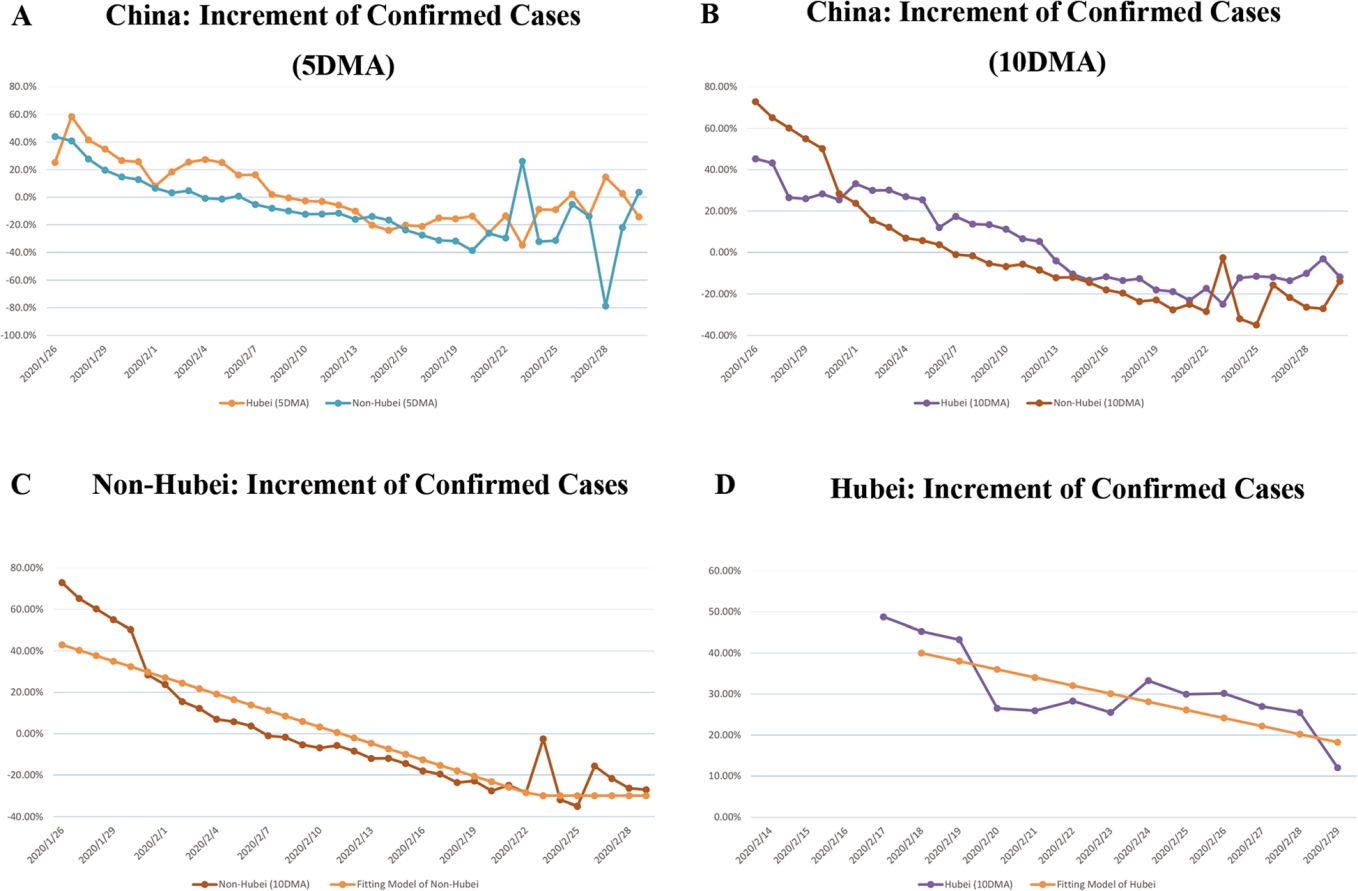
Model construction through China’s experience. **a**, **b** The increment of confirmed cases in Hubei and non-Hubei from Jan 22, 2020 to Mar 1, 2020. **c** The increment and fitting line of confirmed cases in non-Hubei. **d** The increment and fitting line of confirmed cases in Hubei. 5DMA: 5-day moving average; 10DMA: 10-day moving average

As of Mar 5, there were 23,784 confirmed cases, 53,726 cumulative cured cases, 3042 cumulative deaths, 80,552 cumulative confirmed cases, and 670,854 cumulative close contacts in China. Through the analysis, the 5-day moving average (5DMA) and 10-day moving average (10DMA) increment of the confirmed case in Hubei and non-Hubei suggested that the IFP in China was from Feb 6 to Feb 13 (Fig. 3a and b).

Applying the STM model again to establish a 10DMA increment of confirmed cases model in non-Hubei, the fitting line of the trend in non-Hubei could be obtained, which is

$$ y=-0.0387x+1696.2\;\left({\mathrm{R}}^2=0.883\right) $$

(Fig. 3c).

Similarly, in Hubei, the fitting line is

$$ y=-0.022x+965.69\;\left({\mathrm{R}}^2=0.9096\right) $$

(Fig. 3d).

According to the derivatives taken from fitting lines, the epidemic trend in non-Hubei was set as an optimistic scenario with increment of − 3.87%, and the epidemic trend in Hubei as a cautiously optimistic scenario with increment of − 2.20%, and set a relatively pessimistic scenario with increment of − 1.50% (Table 1), which could forecast the situation outside China.

### International epidemic situation and prediction

**Fig. 4:**
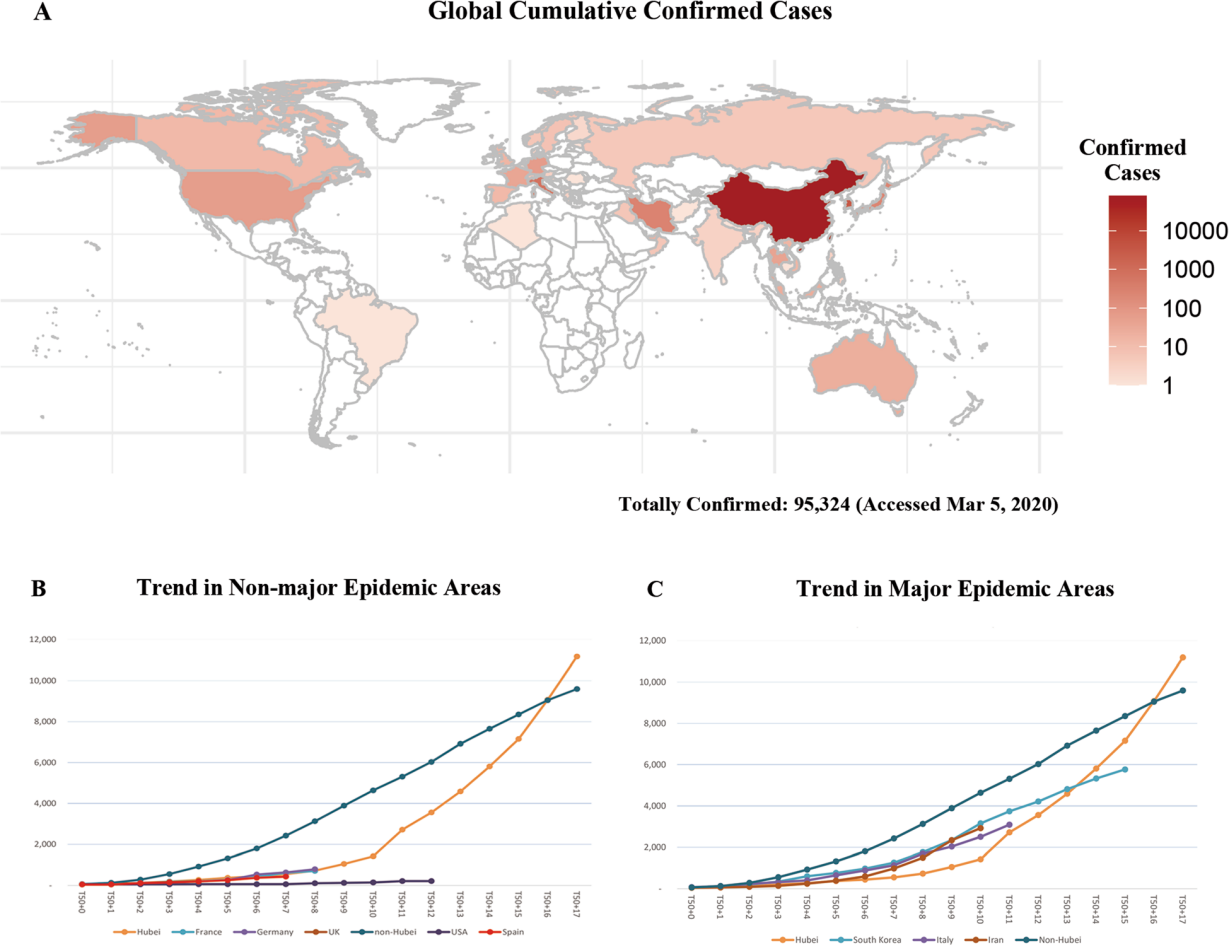
Global epidemic trend. **a** Global distribution of confirmed cases with totally 95,324 cases on Mar 5, 2020. **b**, **c** Comparison of the trends in non-major and major epidemic areas

Data from WHO shows that there were 2232 new cases worldwide on Mar 5, the cumulative number of confirmed cases reached 95,324, and a total of 85 countries have suffered this epidemic (Fig. 4a) [6]. Starting from the cumulative 50 confirmed cases (T50), the cumulative confirmed case trends were compared in different countries with China, and it showed that the trends of France, Germany, United Kingdom, the United States, and Spain stayed steady, while the trends of newly confirmed cases in Korea, Italy, and Iran laid between Hubei and non-Hubei, which have been identified as the major epidemic areas in the globe (Fig. 4b and c).

**Fig. 5:**
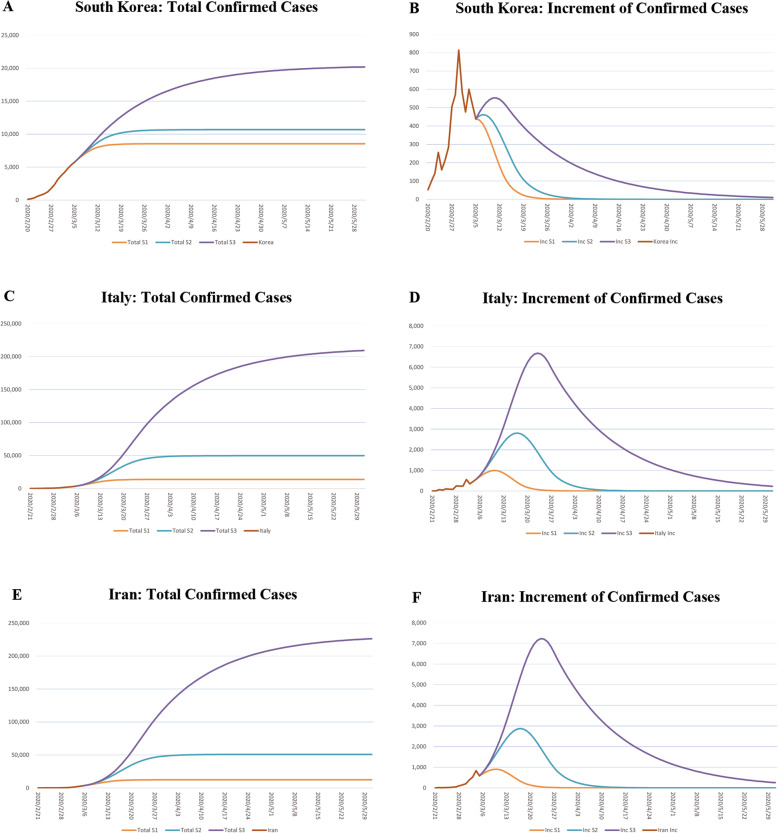
Model application in South Korea, Italy, and Iran. **a**–**f** Predictive total confirmed cases and increment of confirmed cases in South Korean, Italy, and Iran, respectively, with the three scenarios deduced by the State Transition Matrix Model based on the data before Mar 6, 2020

**Fig. 6:**
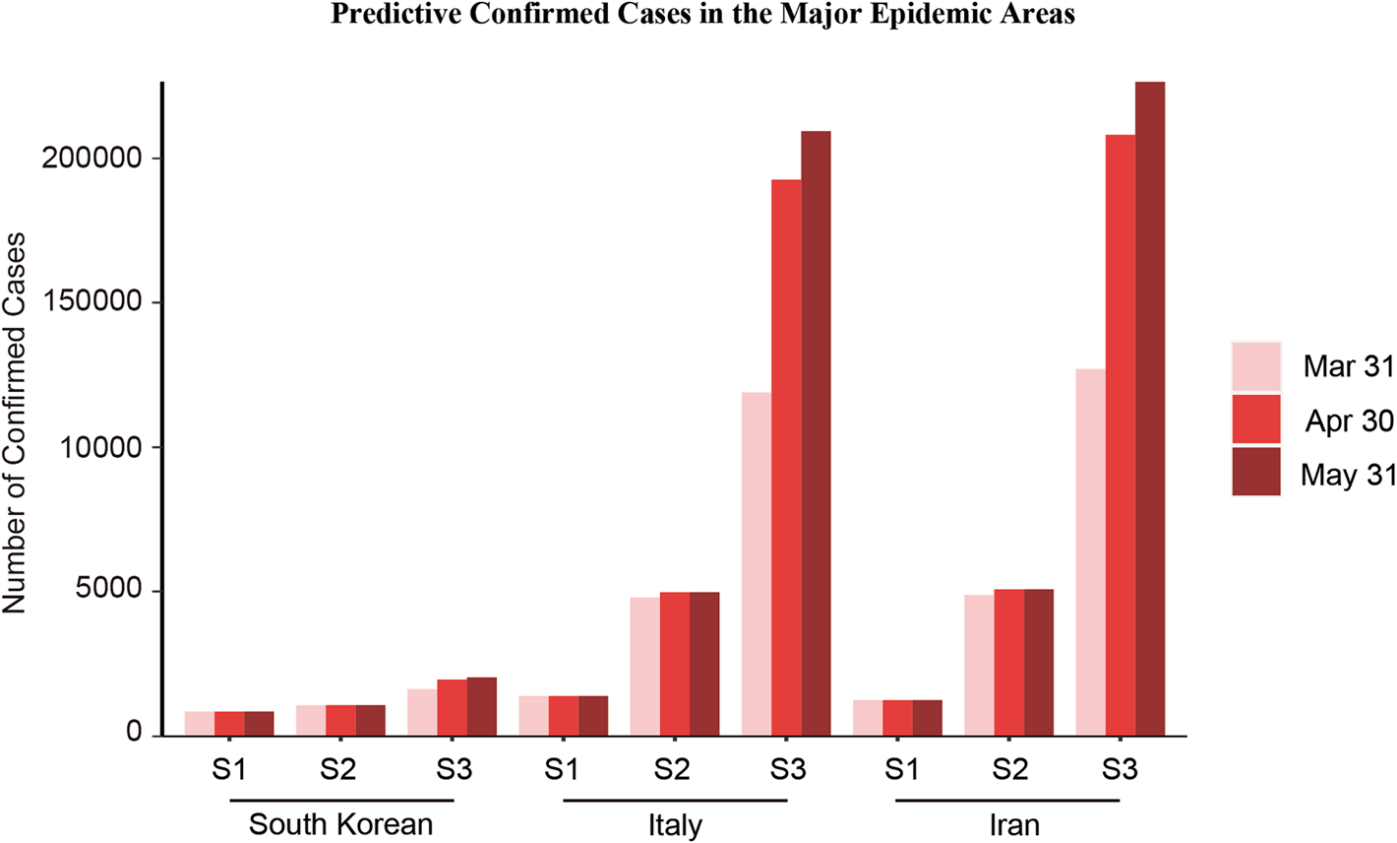
Predictive cumulative confirmed cases in South Korean, Italy, and Iran, respectively, on Mar 31, Apr 30, and May 31. See also Table 4

Then the established STM model was implemented to the three countries. The results showed that the IFP in South Korea would arrive from Mar 6 to 12, 2020 (Fig. 5a and b); the IFP in Italy would arrive from Mar 10 to 24, 2020 (Fig. 5c and d); the IFP in Iran would come from Mar 10 to 24, 2020 (Fig. 5e and f). After completing the model and training set establishment, we compared the cumulative case prediction with the actual data on Mar 6 and Mar 9, which was validation set, and the results overtly testified the efficacy of this prediction model all in Korean, Italy, and Iran (Table 3). By utilizing this model, the approximate number of confirmed cases in the three countries at the end of March, April, and May could be predicted (details show in Fig. 6), which could instruct the international medical resources allocation.

**Table 3:** Training Set and Validation Set of the Epidemic Trends in the Major Epidemic Areas

Countries	South Korea				Italy				Iran			
	Actual	Forecast			Actual	Forecast			Actual	Forecast		
Date		**S1**	**S2**	**S3**		**S1**	**S2**	**S3**		**S1**	**S2**	**S3**
2020/3/5	5766	5766	5766	5766	3089	3089	3089	3089	2922	2922	2922	2922
2020/3/6	6284	6199	6221	6239	3858	3794	3844	3852	3513	3613	3678	3687
2020/3/7	6767	6612	6683	6741	4636	4609	4795	4830	4747	4396	4630	4672
2020/3/8	7134	6990	7142	7268	5883	5515	5965	6064	5823	5250	5806	5921
2020/3/9	7382	7323	7589	7811	7375	6485	7374	7598	6566	6147	7224	7480

S1: optimistic scenario; S2: cautiously optimistic scenario; S3: relatively pessimistic scenario; Inc.: increment

**Table 4:** Predictive Cumulative Confirmed Cases in the Major Epidemic Areas

Country	South Korean			Italy			Iran		
Scenario	S1	S2	S3	S1	S2	S3	S1	S2	S3
2020/3/31	8539	10,651	16,168	13,797	47,930	118,878	12,339	48,847	126,978
2020/4/30	8541	10,695	19,454	13,815	49,797	192,593	12,353	50,795	208,006
2020/5/31	8541	10,695	20,198	13,815	49,802	209,272	12,353	50,800	226,340

S1: optimistic scenario; S2: cautiously optimistic scenario; S3: relatively pessimistic scenario

### The verification of STM model by the data updating after prediction

**Fig. 7:**
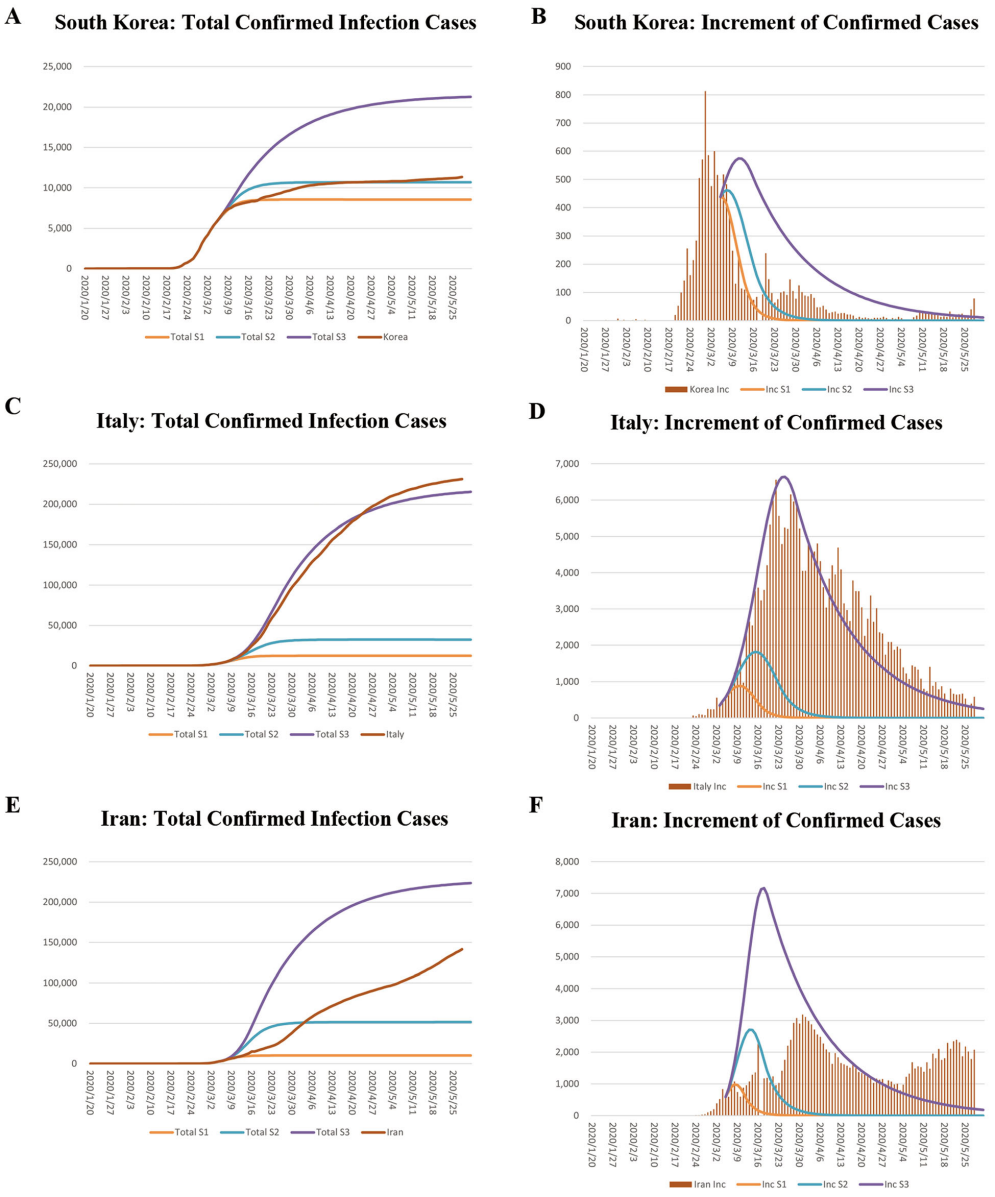
Model verification in South Korea, Italy, and Iran. **a**–**f** Predictive total confirmed cases and increment of confirmed cases in South Korean, Italy, and Iran, respectively, with the three scenarios deduced by the State Transition Matrix Model based on the data before Mar 6, 2020 and the true number of confirmed cases and increment in the three countries

With the time going by almost 3 months, we reran the STM model in South Korea, Italy, and Iran. The results showed that the STM model well predicted the trend in South Korea and Italy, however, not in Iran (Fig. 7a to f). In South Korea, the line of the total confirmed cases laid between the optimistic scenario and cautiously optimistic scenario before April, and fitting the cautiously optimistic scenario after that (Fig. 7a), and so did the IFP of the confirmed cases in South Korea (Fig. 7b). In Italy, the line of the total confirmed cases fitting the relatively pessimistic scenario (Fig. 7c), and so did the IFP of Italy (Fig. 7d). Nevertheless, in Iran, the line of the total confirmed cases laid between the optimistic scenario and the relatively pessimistic scenario (Fig. 7e), and the increment of confirmed cases is still relatively high, which means the IFP of Iran has not come yet (Fig. 7f).

## Discussion

The concept of state transfer matrix was put forward by Russian mathematician Markov. In the early twentieth century Markov found in the early twentieth century that for some factors of a system in the transfer, the result is only affected by the n-1 result, that is, it is only related to the current state, and has nothing to do with the past state. Thus in Markov analysis, the concept of state transition is introduced. The so-called state refers to the state in which objective things may appear or exist; State transition refers to the probability of objective things being transferred from one state to another. In this study, to estimate the IFP of new confirmed cases in the future and the global cumulative case size, we chose markov model cohort simulation. We define the different course states of COVID-19 as state vectors, through which state vectors are used to identify the state of close contacts or patients, and apply line equations to deduce the state transfer equation under N cycles. Using this STM model, we were able to predict the arrival date of IFP for newly confirmed cases in Hubei or non-Hubei provinces, and set three preset scenarios from China to match and fit major endemic areas, including Korea, Italy, and Iran, in order to control model errors.

In this study, the STM model of Korea, Italy and Iran was established in March, and the model data was continuously updated in the background daily. So far, the number of confirmed cases in the world has soared to more than 8 million [15]. As shown in Fig. 7, we successfully predicted the trend of the outbreak in South Korea and Italy, but Iran did not meet expectations. Considering that South Korea and Italy formed good medical experience exchange and medical resources support with Chinese medical experts at the early stage of the outbreak, it is understandable that the model based on Chinese data has a good prediction of the development trend of the epidemic in South Korea and Italy through the absorption of China’s experience and response mode. In the *Lancet*’s the Healthcare Access and Quality Index for 195 Countries and Territories and Selected Subnational Locations, Italy, South Korea and China all reach a good score, which can also be seen as having comparable national healthcare and epidemic prevention and control capabilities. The successful prediction of South Korea and Italy can prove that the development trend of newly and accumulatively diagnosed patients is consistent with the prediction of this model and changes according to our preset prediction model when medical resources are guaranteed and diagnostic capacity is sound. The model in this study did not predict the trend of the outbreak in Iran as expected. The main reason may be that Iran’s diagnostic capacity is limited by the unreasonable allocation of international medical resources, the shortage of PCR kits and the lack of CT scanners. The presence of people with the virus, those with atypical symptoms, and those with mild and asymptomatic symptoms reveals a huge risk of missed diagnosis and recurrence of outbreaks due to inadequate diagnostic conditions [16–18]. In general, the model successfully predicted the outbreak trend of major covid-19 developing countries in the first half of 2020, and had a high guidance effect on the allocation of international medical resources during the epidemic.

Given the punchy transmissibility of COVID-19 [19], isolation and quarantine are undoubtedly the primary options [11]. And currently, predicting models of built for epidemic sprouted out a lot. Ziff et al. established a model of death cases and reported that death cases follow three patterns: exponential growth, power-law behavior, and then exponential decline in the daily rate [20]. Nevertheless, deaths are affected by many factors, such as age [21, 22]. More attention should be paid to the number of new cases, and the rate of increment, attributed to the effect of epidemic prevention and control, can be evaluated to guide the date of return to work. Based on the epidemiological data of 186 county-level administrative units in the UK, Davies et al. established a random inter-compartmental model, in which individuals were divided into susceptibility, exposure, infection (preclinical, clinical, or subclinical) and recovery status (removed from the model). The model is stratified by the age of 5 years, and the impact of various basic interventions on R0 is evaluated [23]. Scheiner et al. adjusted the classic epidemiological model, i.e., SEIR model. It’s based on the transmission characteristics of coronavirus, and concluded that the rule of delay from infection to death was more representative of the actual situation than the classical death dynamics rule, so the traditional SEIR model could be more applicable to the prediction of the transmission of COVID-19 epidemic [24]. Hasan et al. proposed a hybrid model of integrated empirical mode decomposition (EEMD) and artificial neural network (ANN) to predict COVID-19 outbreaks, using window period real-time COVID-19 time series data from 22 January 2020 solstice on 18 May 2020. EEMD is used to decompose the time series data, generate sub-signals, denoise the original data, establish neural network structure to train the de-noised data, and obtain a prediction model superior to the traditional statistical analysis [25]. Tuli et al. use machine learning (ML) and cloud computing to track disease and predict epidemic growth, and deploy an improved model based on MLS on cloud computing platforms to more accurately predict epidemic growth behavior in real time [26].

Moreover, we must strictly follow the coping strategy and learn the Chinese model for dealing with NCP outbreaks. Li et al. developed a simple regression model, and based on this model, they estimated that about 34 founder patients outside of China were not observed in the early stage of transmission, and the global trend approximated an exponential increase, tenfold increase in 19 days [27]. This study reproduced the initial spreading mode to the world, yet made no prediction for the future trend, and exponential growth will be curbed immediately after the attention of local governments, and the IFP will come. Milan Batista proposed an estimate of the final size of the COVID-19 epidemic, the logistic growth model and classic susceptible-infected-recovered dynamic model are used to estimate the final size of the coronavirus epidemic, being approximately 83,700 (±1300) cases and that the peak of the epidemic was on Feb 92,020 [28]. However, as of Mar 5, the number of global cases has reached 95,333, and the IFP for growth in South Korea, Italy, and Iran has not yet arrived, which means the global size will be even more colossal.

Our model is based on the fitting of real data from standard authorities. Through the STM Model, based on data from Hubei and non-Hubei, we predict the IFPs in Korea, Italy, and Iran, while there are still some limitations. Due to the large outbreaks started at different times lines all over the world, the effects of seasonal and geographical factors have not been taken into account. Although the fitting with the Chinese model can better predict the situation around the world, through reference and learning, the response strategies of other countries may be more mature. As China resumes work, the production capacity of various medical resources will gear up rapidly, which will impose a positive impact on the world, and it could be more optimistic that the IFP will come soon.

Local governments, regardless of the speed of outbreaks, should learn from China’s primary response strategy, such as stopping working, reducing gathering, preventing contact transmission, wearing masks, and implementing quarantine. After the NCP being under control, the production and output of medical resources should be intensified, the production of coronavirus detection kits should be accelerated, existing cases should be summarized. More accurate diagnostic criteria should be compiled to prevent massive missed diagnoses in countries lacking the kit. Even if it currently causes some global economic regression, the recovery will swiftly come after holding the throat of NCP and COVID-19.

## Conclusion

Based on data from China, we utilized the State Transition Matrix Model to predict the IFP of disease in countries currently experiencing outbreaks worldwide. If properly controlled, the IFP in South Korea and Italy will come in early March, and the IFP in Iran will come in mid-March. And through almost 3 months, our model fitted well in South Korea and Italy, however, not Iran, partly because of the irrational international medical resource allocation. During this period, countries around the world should work together to fight the epidemic.

## Data Availability

The datasets used and analyzed during the current study are available from the corresponding author on reasonable request.

## Abbreviations

NCP: Novel coronavirus pneumonia
COVID-19: Coronavirus disease 2019
CoV: Coronavirus
SARS: Severe acute respiratory syndrome
ACE2: Angiotensin-converting enzyme 2
IFP: Inflection point
HCHP: The Health Commission of Hubei Province
NHC: The National Health Commission of the People’s Republic of China
WHO: The World Health Organization
STM: State transition matrix
MO: Medical observation
Disc: Discharge
NS: Non-severe case
S: Severe case
Cr: Critical case
Cu: Cured case
D: Death
V: Vector
NCC: New close contacts
5DMA: 5-day moving average
10DMA: 10-day moving average
SEIR: susceptible - exposed - infectious – recovered
EEMD: Empirical mode decomposition
ANN: Artificial neural network
ML: Machine learning
